# AC metrology applications of the Josephson effect

**DOI:** 10.1063/5.0219991

**Published:** 2024

**Authors:** S. P. Benz, J. Biesecker, C. J. Burroughs, M. A. Castellanos-Beltran, P. D. Dresselhaus, N. E. Flowers-Jacobs, A. E. Fox, P. F. Hopkins, R. Johnson-Wilke, D. Olaya, A. Rüfenacht, A. J. Sirois, J. N. Thomas

**Affiliations:** National Institute of Standards and Technology, Boulder, Colorado 80305, USA

## Abstract

The performance of programmable voltage signals that exploit the quantum behavior of superconducting Josephson junctions continues to improve and enhance measurements in metrology, communications, and quantum control. We review advances in pulse-driven digital synthesis techniques with Josephson-junction-based devices. Quantum-based synthesis of voltage waveforms has been demonstrated at frequencies up to 3 GHz and rms amplitudes up to 4 V. Josephson pulse generators have also been used to control and characterize superconducting qubits.

Following the discoveries of helium liquefaction and superconductivity, the Josephson and quantum Hall effects have had the most significant impact on electrical metrology and the redefinition of the international system of units (SI) in 2019.^1^ Robust dc voltage and resistance standard reference instruments (SRIs) based on the Josephson and quantum hall effects were key components in Kibble balance measurements that enabled the role of the last artifact standard, the International Prototype of the Kilogram, to be replaced with a defined value of Planck’s constant *h*.^2^

The quantum behavior of Josephson junctions (JJs) derives from the phase difference between the macroscopic wavefunctions of the Bose–Einstein condensate of Cooper pairs on either side of the junction barrier. This phase difference is periodic in 2*π*, and the time-dependent junction voltage is precisely equal to *h*/(4*πe*) times the rate of change of this phase difference, where *e* is the elementary charge. This relationship creates features unique to Josephson junctions: a junction produces a perfectly quantized voltage pulse with a time-integrated area of *h*/2*e* for every 2*π* change in phase difference. This phase difference oscillates at a “Josephson” frequency *f*_J_ = (2*e*/*h*)*V* that is directly proportional to the junction voltage *V*. The junction generates an ac voltage (or current) at this frequency. Josephson junctions are essentially current-biased voltage-tunable oscillators that can be entrained by microwave bias signals to generate accurate voltages over a range of bias conditions, including dc current, microwave power, and operating temperature. We call each of these ranges the “quantum-locking range” (QLR) of that operating parameter, provided that the phase difference across the junction changes by exactly an integer multiple of 2*π* while the junction precisely produces integer numbers of quantized voltage pulses for every period of the microwave signal.^3^

In this paper, we briefly summarize advances in quantum-based voltage synthesis with superconducting Josephson junctions. We focus on demonstrating voltage waveform synthesis with their quantized voltage pulses and show QLR examples for each. The results presented below will demonstrate that quantum-based synthesis is possible for these frequencies and applications. Synthesis with quantized pulses enables programmable signals and arbitrary waveforms with calculable voltage amplitudes, voltage linearity, and harmonic tones. The citations herein also provide detailed discussions of signal accuracy, especially at a device under test presented with such signals. Signal accuracy requires the characterization of errors such as those from transmission lines, leakage capacitances, and physical properties of the junctions and circuits.

Quantum-based voltage waveform synthesis began with the invention of the programmable Josephson voltage standard (PJVS), which can generate stepwise approximations of sine waves for calibrating ac voltmeters and ac sources. Practical applications began when PJVS output voltages reached 1 V.^4,5^ The PJVS circuit consists of a series array of junctions divided into smaller subarrays that have different numbers *N*_*s*_ of junctions. A continuous-wave microwave frequency signal is applied to the circuit with an amplitude chosen to produce constant voltage steps in each subarray, −*N*_*s*_*hf*/2*e*, 0, or +*N*_*s*_*hf*/2*e*. The PJVS output voltage is programmed by rapidly switching the dc bias current to each subarray to select one of these steps. The PJVS waveforms do not have quantum-based accurate magnitudes at their intended frequencies because the voltage during the transitions between steps is not quantum-based and the voltages of individual steps are only quantum-based after settling on the voltage step.^3,6^ However, differential sampling and subsampling techniques are employed to calibrate voltage sources at frequencies up to 100 kHz.^7,8^

A different technique is used to synthesize voltage waveforms with intrinsically accurate amplitudes. In this case, Josephson junctions are controlled with pulse bias signals, such that a junction generates integer numbers of quantized voltage pulses (typically one) for each bias current pulse for a range of current pulse amplitude. This behavior is employed with digital synthesis and delta-sigma modulation techniques to generate patterns of precisely timed quantized pulses that synthesize single tones or multitone arbitrary waveforms with amplitudes and relative phases that are perfectly calculable. An array of junctions can take a pattern of imperfect (amplitude and phase varying) pulses produced by a conventional semiconductor instrument and convert it into a pattern of perfectly quantized pulses.

The Josephson digital-to-analog converter that uses this technique was first demonstrated in 1995^9,10^ and was later renamed the Josephson arbitrary waveform synthesizer (JAWS).^11^ The signals generated by these JAWS voltage sources have inherent accuracy for amplitude, phase, and linearity due to the quantized pulses and digital synthesis techniques. The first multitone arbitrary waveforms were demonstrated in 2000,^12^ which led to a quantum-based electronic approach to Johnson noise thermometry (JNT) using a quantum-based voltage noise source (QVNS) that synthesized a quantum-accurate comb of constant-amplitude, random-phase, harmonic tones having the same power spectral density as the Johnson noise of a resistor.^13^ Analogous to the measurements of the kilogram that used dc Josephson voltage standards to define Planck’s constant, QVNS JNT measurements were the only electronic and only non-gas-based method used in redefining Boltzmann’s constant.^14^

Superconducting Josephson pulse generator (JPG) circuits have also been designed to produce a many-fold larger magnitude pulse with arrays of 500 and 4650 Josephson junctions. The JPGs have been used to control and characterize superconducting qubits.^15,16^ JPGs provide accurate, reproducible signals that do not require calibration, unlike room-temperature-generated signals. This feature has the potential to enhance the scalability of superconducting quantum processors. In this Letter, we will describe the state-of-the-art performance of three different JAWS circuits that were developed for ac voltage and RF metrology up to gigahertz frequencies and the qubit control measurements performed with a JPG. We will describe the latest measurements with these devices while emphasizing the quantum locking ranges of circuit bias parameters that demonstrate their unique quantum-based character.

The Josephson junctions in JAWS circuits are driven by a sequence of current pulses; by controlling the density, timing, and polarity of the quantized pulses generated by the JJs, JAWS systems produce intrinsically accurate sine wave voltages. JAWS systems have been implemented for ac voltage metrology at “audio-range” frequencies below 20 kHz and rms voltages up to 4V. Figure 1 shows (a) the spectrum and (b) the QLR and total harmonic distortion (THD) results for the highest reported 4V quantum-based synthesized signal. The signal was generated using 202 480 JJs divided between two superconducting-integrated circuit chips that are co-located on the 4K stage of a cryocooler. The results were presented at the 2018 Conference on Precision Electromagnetic Measurements^17,18^ but are published here.

Figure 1(a) shows the measured spectrum (FFT of the measured waveform integrated over 1mA dc offset dither current) for a JAWS-synthesized 4V rms, 1 kHz signal; nonlinearities of the measuring analog-to-digital converter (ADC) generate the harmonic distortion observed at higher frequencies. Figure 1(b) shows the voltage residuals of a fit to a sinusoid vs dc offset and waveform period for the 1 kHz, 4V signal. The voltage QLR is the central, nearly zero voltage band between (−0.9 mA and +0.9 mA) dc offset current; the vertical stripes at various period values reveal digitizer distortion. The overlain green data (scale on the top axis) show the THD QLR vs dc offset current. The THD is calculated from the voltage residuals at each current. The voltage residuals and THD are independent of dc offset current over the same 1.8 mA QLR. When the JAWS circuits are biased slightly out-side the QLR range, an increase or decrease in residual voltage and an increase in THD occurs. This is due to the presence or absence of desired JJ pulses, which in turn distorts the perfectly synthesized sine wave, thus changing the magnitude of the 1 kHz fundamental and generating harmonic distortion that adds to that of the digitizer.

One main application of JAWS systems is to establish ac voltage direct traceability to the SI without the need for a chain of calibrations. Direct comparisons between intrinsically accurate JAWS signals and those of other ac sources are a paradigm shift for ac voltage calibrations that are typically performed with rms detectors. JAWS voltage waveforms have been used as references to calibrate transfer standards and other instruments.^19–27^ Additionally, calibration with JAWS standards typically reduces overall measurement duration and measurement uncertainty while eliminating the dependence on artifact standards and other calibration chains.

JAWS systems have also been used in two-terminal and four-terminal automated impedance bridges for precise determination of resistor and/or capacitor ratios.^28,29^ A fully digital bridge has been developed that uses two independent JAWS sources to generate accurate arbitrary voltages to measure any complex impedance ratio. Measurements using this type of bridge have demonstrated that a comparison of any two impedances is feasible over a large frequency range with uncertainty below 0.5 *μ*Ω/Ω.^28^ Historically, high-precision bridges are manually operated and exhibit significant limitations in terms of impedance value and type due to the use of transformers as the source. The use of JAWS systems with a digital bridge eliminates the need for numerous individually calibrated transformers for each ratio and phase.

For frequencies above 1 MHz, diplexer JAWS circuits are used to better match the output impedance to 50 Ω.^30^ Diplexers terminate the low-impedance of a long JJ array, thereby reducing on-chip signal reflections and standing waves when transmitting higher-frequency waveforms from the cryogenic JAWS to a room-temperature device under test (DUT). An integrated superconducting diplexer is implemented at the JJ array output of the Very-High-Frequency-band JAWS (VHF-JAWS), which NIST is developing as a quantum-based source for RF power calibrations.^31^

The latest VHF-JAWS circuits have demonstrated signal frequencies up to 50.05 MHz with substantial QLR (1 mA dc offset) at the maximum open-circuit voltage of 50 mV (−19 dBm into 50 Ω). Since the synthesized voltage signals are programmable and calculable, and have perfect linearity in magnitude, they have been used to measure the nonlinearity (and harmonic distortion) of VHF digitizers and power sensors.^32^ Vertical bars in Fig. 2 indicate the boundaries of the dc-bias QLRs for VHF-JAWS signals at eight frequencies up to 50.05 MHz, measured using a high-speed digitizer. The results confirm that the JJs continue to produce quantized signals as frequency increases. Still, some signal loss due to impedance mismatch is unavoidable at radio frequencies. The frequency dependence of the voltage magnitude within the QLRs shown in Fig. 2 demonstrates the non-ideal transfer function to the DUT. The next circuit-design iteration of VHF-JAWS will aim to further minimize mismatches to reduce the impact of the RF transfer function. Additionally, performing an RF calibration to shift the measurement plane to the JJ array is necessary for practical calibrations.

At even higher frequencies, RF-JAWS circuits synthesize signals up to 3 GHz with amplitudes up to 22 mV. Operating at these higher frequencies requires changing both the measurement procedure and the digital synthesis technique used to generate the bias pulse pattern. To remove the transfer function effect of the cryogenic cabling, a cryogenic probe station and a vector network analyzer (VNA) are used to perform a large signal network analyzer (LSNA) calibration of the system and move the reference plane of the measurement to the JJ array circuit. The NIST researchers reduced the JJ circuit to its simplest element: a series array of 1500 JJs embedded in a 50 Ω coplanar wave-guide with ground-signal-ground (GSG) probe launches on both ends of the array. This system is used to compare RF-JAWS signals with a classical power calibration at a −48 dBm into the 50 Ω VNA at frequencies up to 3 GHz.^33–35^

JAWS systems at all frequencies use a delta-sigma algorithm to determine a JJ pulse pattern, which generates a desired waveform in a bandwidth that is much smaller than the maximum pulse repetition rate.^36^ The typical JAWS pulse repetition rate of around 15 GHz implies that audio frequency JAWS signals can use a low-pass delta-sigma modulator algorithm where the bandwidth with low digitization noise is centered at dc. On-chip inductors, twisted-pair output leads, and high-impedance DUTs then naturally low-pass filter the JJ array output, removing digitization harmonics reaching the DUT. VHF-JAWS waveforms operate at the edge of the regime where a low-pass delta-sigma algorithm gives acceptable performance, though explicit diplexers are now needed to filter the JJ output signal. However, RF-JAWS operates in a regime where a bandpass delta-sigma algorithm is required, since there is only a relatively narrow bandwidth, typically (10–100) MHz, within which the desired waveform resides and the unwanted digitization harmonic signals are excluded.^37,38^ The out-of-band digitization harmonics can be a problem for some broadband applications but are acceptable for measurements with a VNA that has excellent frequency discrimination.

In RF-JAWS, QLR measurements are still used to confirm that the system is operating correctly. However, THD measurements are not practical for determining QLR with a bandpass delta-sigma algorithm. Instead, we measure the magnitude of the desired waveform and the integrated in-band noise power.^39,40^ A consistently missing or extra pulse in the pattern is equivalent to adding a frequency comb to the spectrum of the calculated pattern, with the phase of the comb frequencies determined by the location of the missing or extra pulse in the pattern. The effect on the magnitude of a single-frequency waveform, therefore, depends on the phase of that comb line, but the appearance of comb frequencies in the surrounding bandwidth will increase the total in-band noise power.

Measurements of waveform magnitude and noise are plotted in Fig. 3(a) as a function of dc offset bias current through the JJ array when generating a tone at 500 MHz. The dc current QLR is about 2 mA. We typically perform this QLR at each waveform frequency [density plot of total noise power in Fig. 3(b)] and observe only small changes in the quantum locking range as a function of synthesized waveform frequency. We observe an agreement of 0.1 dB between the calculated quantum-based RF-JAWS output and a traceable LSNA-calibrated measurement at frequencies below 500 MHz.^35^ We have also made uncalibrated measurements of more complicated modulated waveforms: a 101-tone waveform in a 40 MHz bandwidth centered at 1.005 GHz and a 10 MHz quadrature-phase shift-keying (QPSK) waveform on a 1.005 GHz carrier.^41^

Further increases in JAWS signal frequencies will require faster JJs and faster, narrower bias pulses, because the width of the quantized pulses should be much smaller than the clock frequency. The finite width of the quantized pulses had a measurable effect on the magnitude of synthesized 3 GHz signals.^35^ The quantized pulse width, which is inversely proportional to the JJ critical current and its normal resistance, is typically about 50 ps for our JAWS JJs. Faster JJs have been created in the context of digital superconducting single-flux-quantum computing.^42^ However, making use of faster JJs requires faster and narrower bias pulses. One approach to decreasing the width of bias pulses is to use fast optical pulses and fast photodiodes.^43–46^

In contrast to traditional semiconductor electronics (TSCE) that operate at room temperature, cryogenic single-flux-quantum (SFQ) electronics are an alternative method for measuring and controlling qubits and have the potential to drive qubits with calculable, reproducible pulse signals and address scalability challenges of cryogenic quantum processors. Recent advancements have demonstrated the capability of SFQ circuits to control superconducting qubits.^47,48^ However, experiments in which the SFQ electronics were collocated nearby the qubits encountered issues of decoherence and loss attributed to quasiparticle poisoning.^15^ By relocating the pulse generation device from the millikelvin stage to the 3 K stage, we ameliorated these challenges and demonstrated high-fidelity single-qubit gates while preserving key qubit characteristics such as lifetime (T_1_) and coherence time $\left({\text{T}}_{2}^{*}\right)$.^15,16^

Like the above JAWS circuits, our Josephson pulse generator (JPG) circuit consists of an *N*-junction series array to produce quantized pulses with *N*-fold-higher amplitude. The JPG is different from a JAWS array in that it is designed to operate over a broader range of temperatures (using different junction barriers) and is used to generate finite trains of periodic quantized N-fold pulses rather than arbitrary analog waveforms. Thus, the train of pulses sent by the JPG is not periodic and does not correspond to delta-sigma codes. The number of junctions in the JPG array operating at 3 K is selected to ensure that sufficient pulse amplitude reaches the qubit at the 10 mK stage after the signal passes through the intermediate attenuating coaxial signal lines. Our latest device consisted of a 500-JJ array.^16^ We validated the pulse magnitude by measuring the 0.9 mA QLR of the dc bias current (*I*_*b*_) of the constant voltage step in the current–voltage curve, Fig. 4(a). Digital control of the qubit and precise qubit state preparation was achieved with the JPG generating one *N*-fold-amplitude pulse per clock period. The exact number of sequential pulses sent to the qubit depended on parameters such as coupling capacitance to the qubit and the total attenuation in the lines between the 3 K and the 10 mK stages of the fridge. By adjusting the clock phase, the JPG can perform arbitrary two-axes rotations of the qubit on the Bloch sphere.^15^

We observed the same qubit lifetime and coherence times, T_1_ and ${\text{T}}_{2}^{*}$, when it was controlled with JPG signals as with TSCE signals. We also performed randomized benchmarking^49^ of single-qubit gates and achieved agreement within 10% compared to our TSCE control system. Interleaved randomized benchmarking on individual JPG gates revealed an average gate error of 0.46%, indicating improved gate fidelity compared to prior efforts and highlighting the potential of a 3 K-operated Josephson microwave source for scalable qubit control.

Through optimized JPG–qubit coupling, we nearly achieved coherence-limited digital control with a gate fidelity of 99.54% using randomized benchmarking, consistent with individual gate fidelities obtained from interleaved randomized benchmarking. Potential improvements may be achieved from longer coherence times, faster gates, higher anharmonicity qubits, or faster pulse generators operating above qubit frequencies with variable pulse-delivery timing.^50^

JAWS systems with arrays of Josephson junctions have realized quantum-based voltage waveform synthesis for signal frequencies up to 3 GHz. Synthesized waveforms that exploit the pulse quantization of junctions are being applied to ac and dc voltage metrology, RF communications, and qubit control, and quantum locking range measurements of bias parameters can be used to verify they are realized with quantized pulses. Signals with higher frequencies and larger amplitudes are possible with appropriate circuit designs as well as faster junctions and bias electronics.

## Figures and Tables

**FIG. 1.**
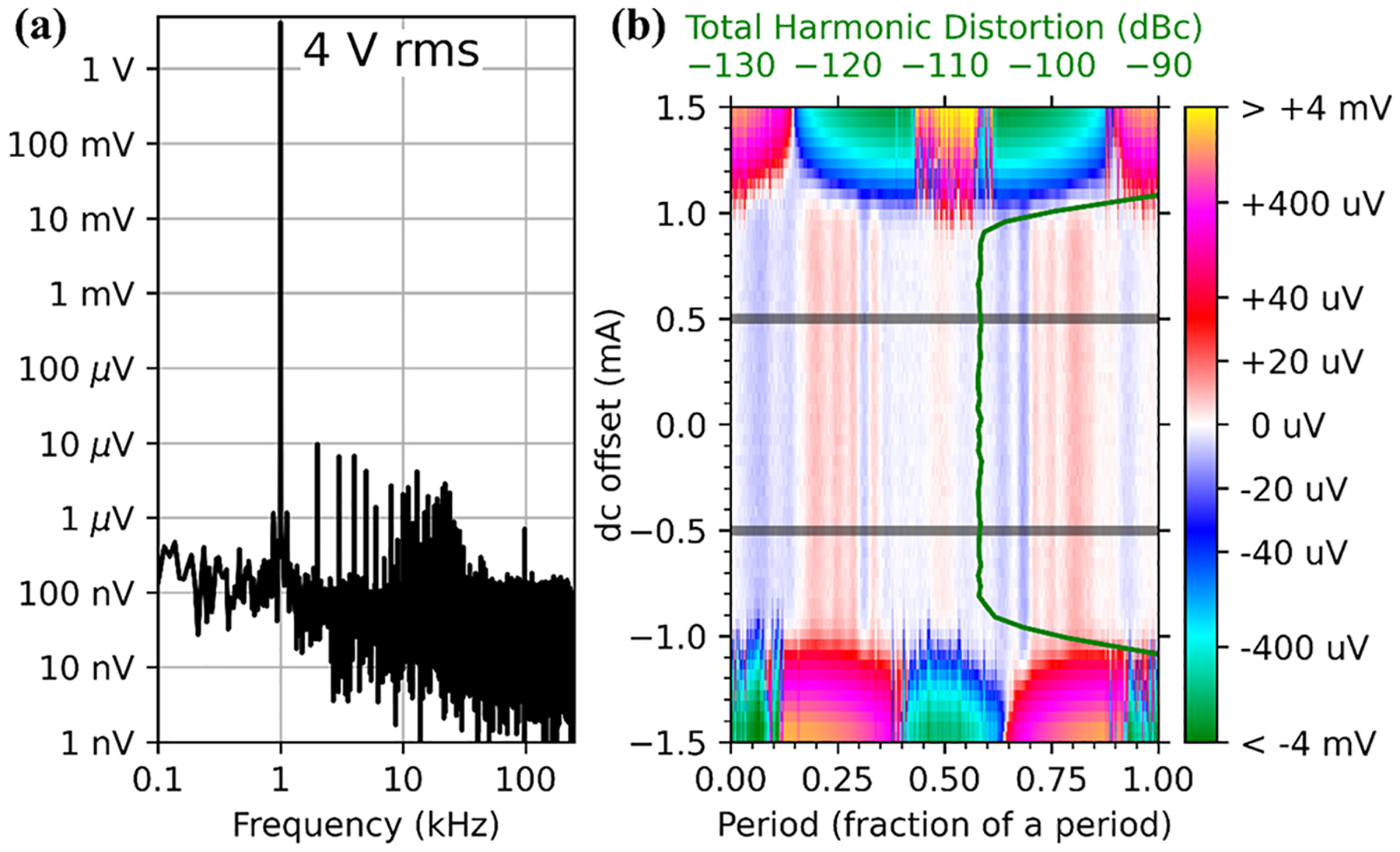
Measurements of a 1 kHz JAWS-synthesized sine wave with a 4 V rms magnitude. The measured spectrum (a) shows the 1 kHz fundamental and harmonic distortion produced by the measurement ADC. In (b) are plotted the voltage residuals (color bar) and THD (green, top axis) vs dc offset bias current (and waveform period for residuals). The horizontal gray lines indicate a target 1 mA QLR.

**FIG. 2.**
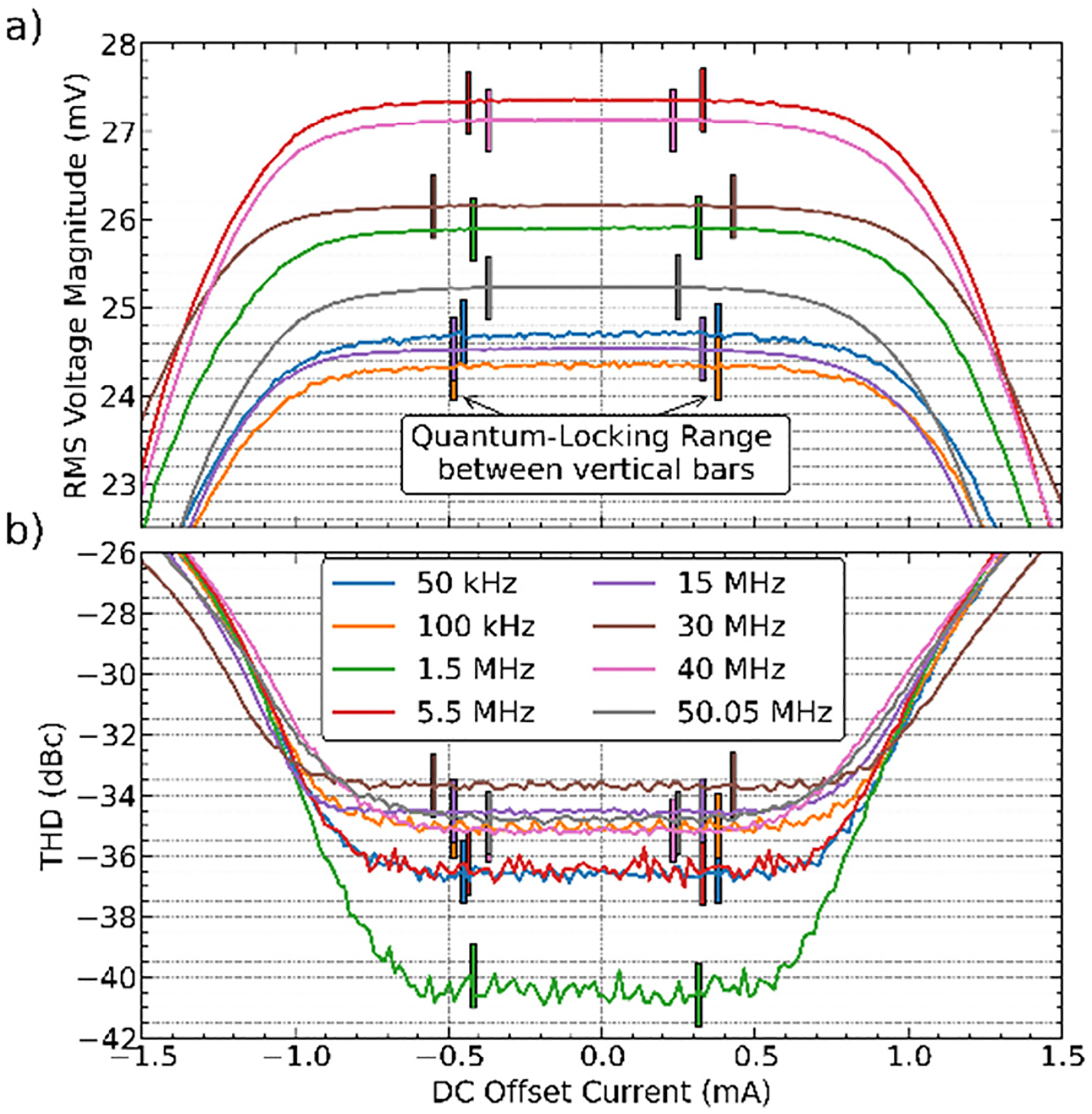
(a) RMS voltage and (b) THD vs DC offset current for VHF-JAWS.^31,32^ A single JJ array generates single-tone waveforms each with 50 mV open-circuit rms voltage for 8 frequencies between 50 kHz and 50.05 MHz. The 30 MHz tone in (a) is offset by +6.5 mV for visual clarity.

**FIG. 3.**
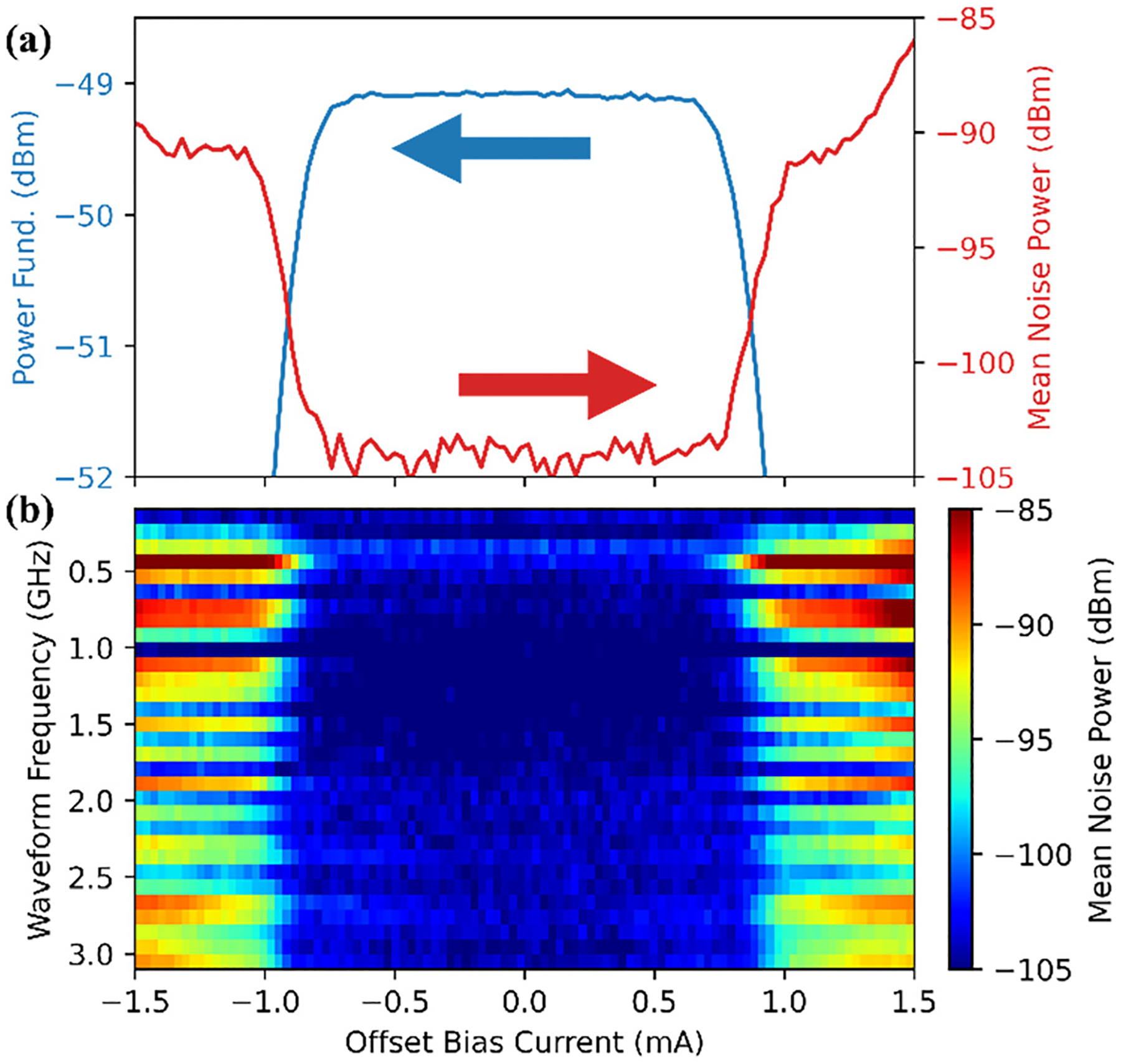
(a) DC offset bias current quantum locking range measurements showing the power at the fundamental of a 500 MHz tone (blue) and the mean noise power surrounding the tone (red). (b) Similar measurements are performed at each waveform frequency (density plot) showing that the system is behaving correctly when generating these RF waveforms up to 3 GHz with a dc current QLR of almost 2 mA.

**FIG. 4.**
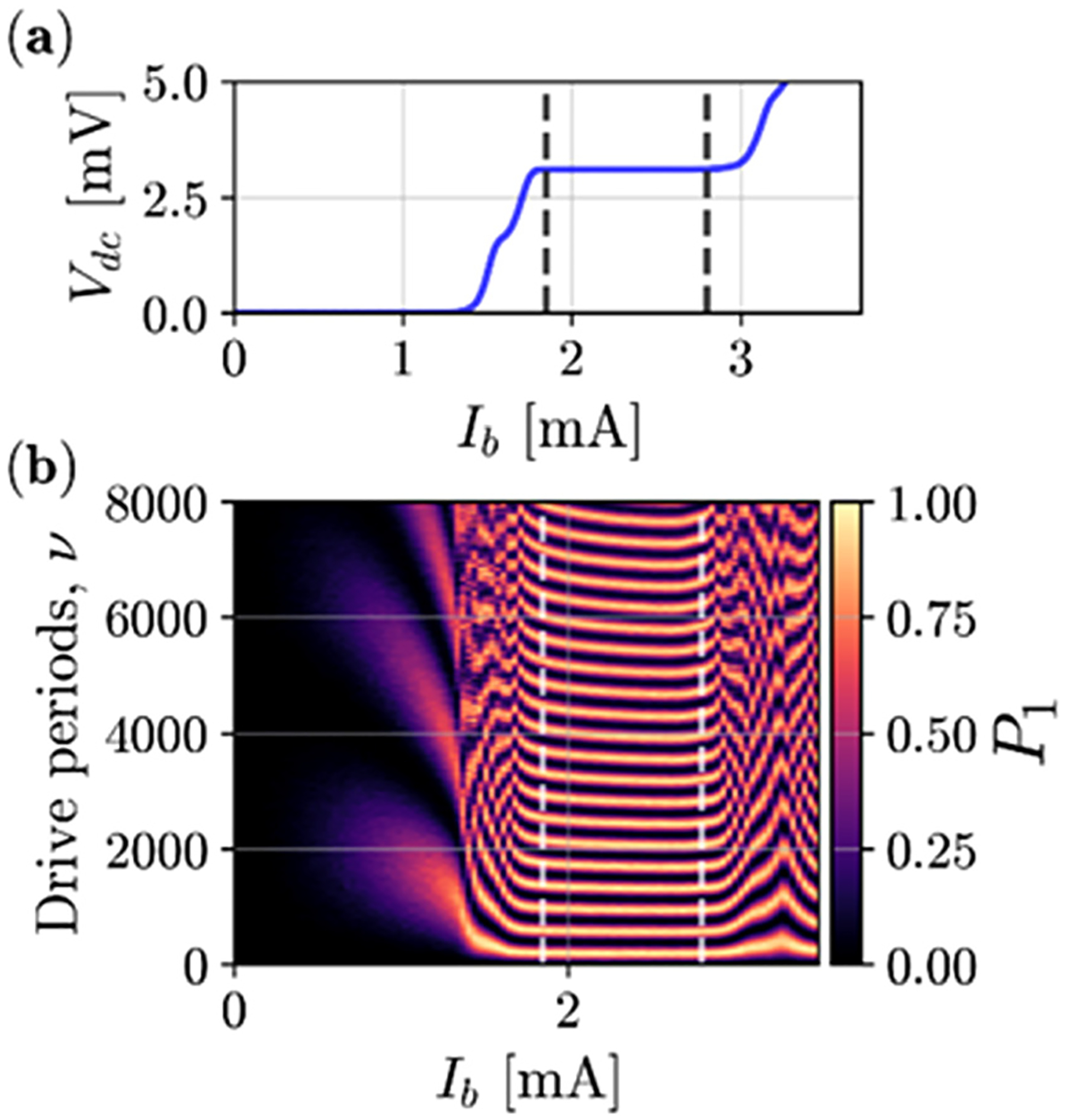
(a) Voltage–current characteristic of a 500-JJ JPG biased with a 3 GHz signal.^16^ (b) Rabi oscillations of the qubit biased with the JPG signal as a function of the bias current and number of drive periods. The color bar (*P*_*1*_) is the probability of the population in the first excited state. Dashed lines indicate the bias current QLR where a constant Rabi frequency is generated.

## Data Availability

The data that support the findings of this study are openly available in National Institute of Standards and Technology, at https://doi.org/10.18434/mds2-3254, Ref. 51.
